# The Modified Bosniak Classification for Intermediate and High-Risk Renal Cysts

**DOI:** 10.7759/cureus.37331

**Published:** 2023-04-09

**Authors:** Khalid Alkhamis, Omai Alsasi, Mohammed Alzahrani

**Affiliations:** 1 Radiodiagnostics and Medical Imaging Department, Prince Sultan Military Medical City, Riyadh, SAU

**Keywords:** pediatric mri, pediatric renal cysts, renal ultrasound, bosniak 3/4 cyst, kidney ultrasound, renal cyst, pediatric, mbosniak, modified bosniak classification

## Abstract

Background

Renal cysts are uncommon among the pediatric population, and their transformation into malignant lesions is also uncommon. Early detection can prevent further complications and protect renal function. Bosniak classification is a computed tomography-based classification for renal cysts developed for adults. Children are more susceptible to CT radiation. Therefore, a modified Bosniak classification for children based on the ultrasound (US) can be used if it shows reliability and accuracy.

Aim

To apply the modified Bosniak classification system among children with renal cysts.

Methods

This was a retrospective study that was conducted on pediatric patients who underwent surgery for intermediate and high-risk complex renal cysts in Prince Sultan Military Medical City, Riyadh, Saudi Arabia using radiological information from 2009 to 2022. The collected data included demographics, medical history, radiological findings, and characteristics of renal cysts. SPSS Statistics v. 22 (IBM Corp., Armonk, NY) was used to analyze the data.

Results

There were 40 children included in the study based on the US-modified Bosniak classification. Around 26.3% of patients had class I and 39.5% had class II renal cysts. Histopathology showed that 10% had Wilms tumor, and 15% had benign lesions. There were significant correlations between pathology findings and US findings (p=0.004), and CT findings (p=0.016).

Conclusion

The modified Bosniak classification based on the US is sensitive, specific, and sufficiently accurate in the classification of renal cysts among children. Also, the size of the renal cysts can be a diagnostic marker of differentiation of benign and malignant cysts with high sensitivity and specificity.

## Introduction

Renal cysts are fluid-filled epithelial lined structures arising from dilation in any part of the collecting duct or nephron [1]. The formation of renal cysts can be observed in different kidney diseases; most of these diseases are genetically transmitted, whereas some of them are acquired or developmental [2]. Cystic renal diseases can be either hereditary or non-hereditary; the former involves ciliopathies, tuberous sclerosis, and nephronophthisis, whereas the latter involves simple renal cysts, complex renal cysts, and multicystic dysplastic kidney [3]. In children, most renal cysts are related to hereditary disease rather than acquired cystic diseases or simple cysts, as seen in adults [4]. In hereditary cystic kidney diseases, defects in the epithelial cilia function or structure of the kidney have been implicated in cyst formation [2].

The prevalence of renal cystic disease was estimated to be 4.81% in the Arabian Gulf countries [5]. Renal cystic diseases can occur in infancy, childhood, and adolescence. The overall incidence of renal cystic diseases varies from 0.44 cases/10000 births for neonatal-onset genetic polycystic kidney diseases to 4.1 cases/10000 births for sporadic kidney cystic diseases [6]. In the pediatric population, kidney cysts can be simple cortical cysts, and they may involve a large group of diseases that can cause further complications, such as proteinuria and chronic kidney disease [2]. The role of imaging, especially ultrasound (US), is to perform a diagnosis in the least invasive way for young patients [7].

The US is the cornerstone of imaging cystic diseases. A high-frequency transducer should be used for a detailed examination of the cystic kidney [8]. Magnetic resonance imaging (MRI) is not always required; rather, it is used very sparingly; its usage is involved in ruling out malignancy in complex cysts [4]. Computed tomography (CT) can be used as an alternative to MRI in case of the unavailability of MRI or among claustrophobic patients. CT is also useful in patients with complicated cysts [4].

In earlier years, kidney cysts were classified according to their morphological features, whereas in recent years, radiological, clinical, and genetic characteristics are involved in the classification [2]. For more than 25 years, the Bosniak classification system has been used for categorizing complex cystic masses in the kidney [9]. This classification system was based on computed tomography (CT), and it divides renal cysts into five categories based on their imaging features on CT. In literature, this system is proposed as a method to separate benign from malignant cystic lesions in the kidney [9-11].

The Bosniak classification stratifies the risk of malignancy in cystic renal masses [12]. Benign lesions involve categories I and II, whereas category IIF is suspicious for malignancy and requires follow-up. Categories III and IV refer to potentially malignant and predominantly malignant lesions, respectively. Many institutions tended to use non-radiation modalities such as ultrasonography (US) and magnetic resonance imaging (MRI) instead of CT to avoid multiple exposures to radiation, especially for patients who require follow-up [13]. The Bosniak classification was originally developed for classifying renal cysts in adults. However, it was suggested that this US-based modified Bosniak classification is reliable enough to classify renal cysts in children [14,15]. Modified Bosniak classification offers criteria to diagnose simple and complex renal cysts, which assist in the follow-up and management of pediatric patients [16].

## Materials and methods

This is a retrospective study that is conducted on pediatric patients who underwent surgery for intermediate and high-risk complex renal cysts by utilizing the radiology information system (Centricity®, Centricity Wealth Tech, Delhi, India) database in Prince Sultan Military Medical City, Riyadh, Saudi Arabia. Low-risk patients with simple cysts were excluded. Patients who had complex renal cysts due to trauma or previous surgical intervention were also excluded. Medical and imaging records of pediatric patients from 1 January 2009 to 31 December 2022 were reviewed. The collected data included the demographics of patients, including the age, sex, medical history, US, CT, and MRI findings, and characteristics such as size, border, wall thickness, septations, irregularity of septations, vascularity, solid components, and calcifications. According to these findings, each case was assigned one of the four categories as defined in the modified Bosniak classification [17]. One pediatric radiologist interpreted the data and a more senior pediatric radiologist reviewed the results and then assigned each case to one of the four modified Bosniak classification categories. Both radiologists agreed about the assigned category and no inter-observer difference was found. A comparison was made between the binary cohort of the Bosniak categories (I and II vs. III and IV) and the histological results. Regarding diagnostic accuracy, benign versus intermediate-risk lesions were calculated for each imaging modality. Benign features include simple/benign cysts, chronic pyelonephritis, and renal dysplasia, whereas intermediate features include cystic nephroblastoma, metanephric adenoma, and multilocular cystic nephroma.

## Results

A total of 40 children were included in our study, half of the children were in the age range of 1-10 years, and females were more dominant compared to males; 23 (57.5%) vs. 17 (42.5%) for females and males, respectively (Table 1).

**Table 1:** Demographics of children

		Count	Percentage
Age	<1 year	6	15.0%
	<1month	7	17.5%
	>10 years	7	17.5%
	1-10 years	20	50.0%
Sex	Male	17	42.5%
	Female	23	57.5%

The characteristics of renal lesions are shown in Table 2. The median size of the lesions was 3.1 cm, and the median of wall thickness was 0.1 cm; 37 (94.9%) patients had lesions with well-defined borders, and irregularity of septation was found in nine (30%) patients. There were 27 (67.5%) patients who had lesions with internal septations, and 24 (63.2%) patients had lesions with no internal vascularity in color Doppler. More than one-half of patients, 27 (67.5%) patients, did not have lesions with solid components, and 34 (85%) patients had lesions without calcifications.

**Table 2:** Characteristics of renal lesions

Characteristics of renal lesions		Median	25th percentile
Size		3.1 cm	1.3-7
Wall thickness		0.1 cm	0.1-0.3
Border	Well define	37	94.9%
	Lobulated	1	2.6%
	Micro lobulated	1	2.6%
Irregularity of septations	No	21	70.0%
	Yes	9	30.0%
Septations	No	13	32.5%
	Yes	27	67.5%
Vascularity	Absent	24	63.2%
	Peripheral	2	5.3%
	Rim	8	21.1%
	Solid	4	10.5%
Solid components	No	27	67.5%
	Yes	13	32.5%
Calcifications	No	34	85.0%
	Yes	6	15.0%

The findings of imaging and histology are shown in Table 3. Based on US-modified Bosniak classification, more than one-half of lesions were classified as class I (10; 26.3%) or class II (15; 39.5%), whereas based on CT-modified Bosniak, the first and second classes were less reported; two (10.5%), and six (31.6%) belonged to class I and II, respectively. According to MRI-modified Bosniak, there were only three (37.5%), and two (25%) belonged to classes I and II, respectively. Histopathology was performed for only 10 samples, and six (15%) of them were benign lesions, whereas four (10%) were Wilms tumors.

**Table 3:** Imaging and histopathology findings Abbreviations: mBosniak, modified Bosniak

		Count	Percentage
US mBosniak	I	10	26.3%
	II	15	39.5%
	III	4	10.5%
	IV	9	23.7%
CT mBosniak	I	2	10.5%
	II	6	31.6%
	III	3	15.8%
	IV	8	42.1%
MRI mBosniak	I	3	37.5%
	II	2	25.0%
	III	1	12.5%
	IV	2	25.0%
Histopathology	Not done	30	75.0%
	Wilms tumor	4	10.0%
	Benign lesion	6	15.0%

**Figure 1:**
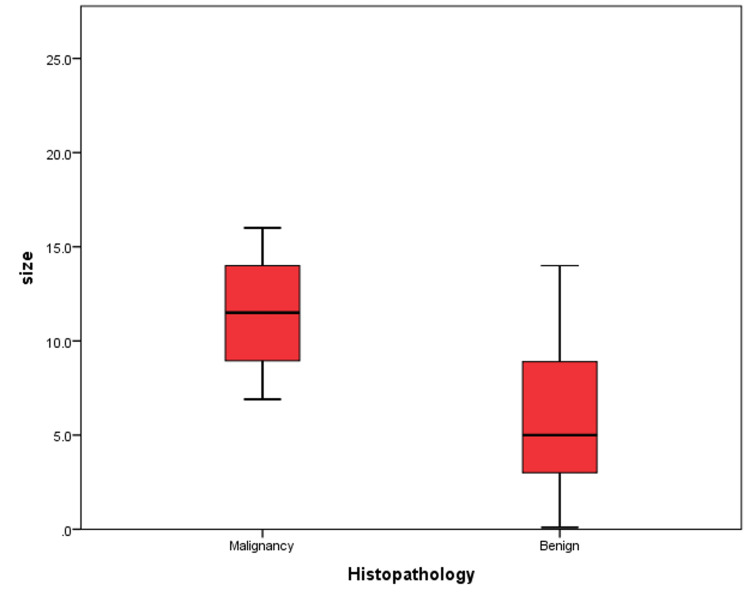
The correlation between histopathology and size of lesions

**Figure 2:**
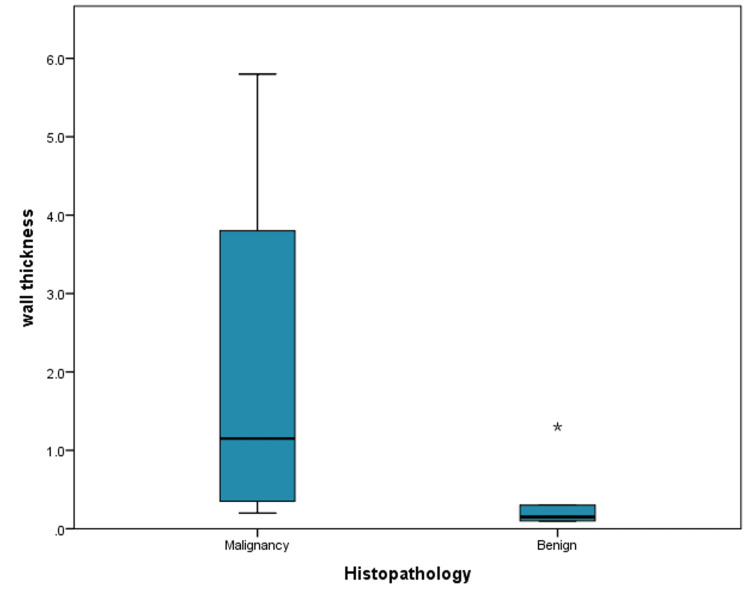
The correlation between histopathology and wall thickness of lesions

The correlations between histopathology findings and different variables are shown in Table 4. There was a significant correlation between the size of lesions and histopathology (p=0.018) (Figure 1). Also, there was a significant correlation between histopathology findings and the wall thickness of the lesions (p=0.005) (Figure 2). A significant association was found between the irregularity of septations and histopathology findings (p=0.026). The presence and absence of solid components were significantly associated with histopathology findings (p=0.009).

**Table 4:** Association between imaging characters and histopathology

			Histopathology							
			Malignancy			Benign			p-value	
Size		Median, IQR	11.5 cm	14	5 cm		3-8.9	0.018		
Wall thickness		Median, IQR	1.2 cm	0.4-3.8	0.2 cm		0.1-0.3	0.005		
Border	No lesion	N, %	0	0.00%	0		0.00%	0.055		
	Well defined	N, %	3	75.00%	5		100.00%			
	Lobulated	N, %	0	0.00%	0		0.00%			
	Micro lobulated	N, %	1	25.00%	0		0.00%			
Irregularity of septations	No	N, %	0	0.00%	3		50.00%	0.026		
	Yes	N, %	2	100.00%	3		50.00%			
Septations	No	N, %	2	50.00%	1		16.70%	0.534		
	Yes	N, %	2	50.00%	5		83.30%			
Vascularity	No lesion	N, %	0	0.00%	3		60.00%	0.137		
	Peripheral	N, %	1	25.00%	0		0.00%			
	Rim	N, %	2	50.00%	1		20.00%			
	Solid	N, %	1	25.00%	1		20.00%			
Solid components	No	N, %	0	0.00%	4		66.70%	0.009		
	Yes	N, %	4	100.00%	2		33.30%			
Calcifications	No	N, %	3	75.00%	6		100.00%	0.487		
	Yes	N, %	1	25.00%	0		0.00%			

**Figure 3:**
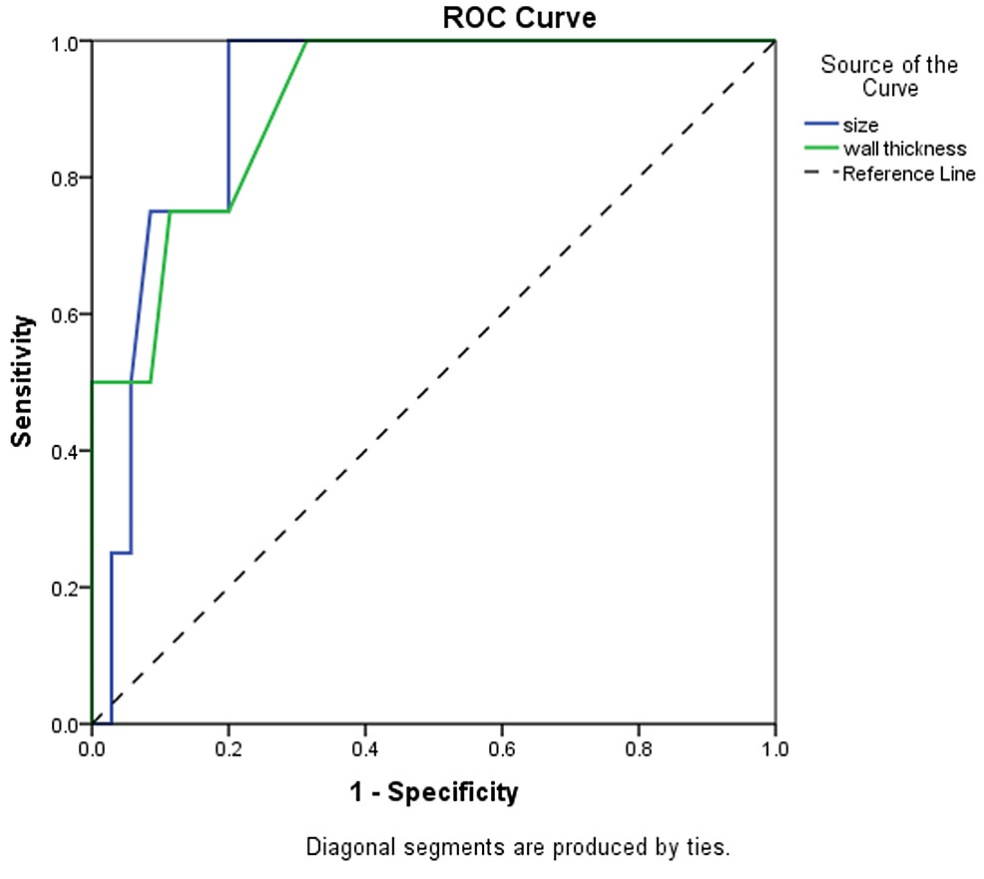
Receiver operating characteristic curve for size and wall thickness

The diagnostic characteristics of the size and wall thickness of lesions were investigated (Table 5). The AUC of size was the same as wall thickness (AUC=0.911), with the same significance (P=0.008) at a size of 6.5 cm and a wall thickness of 0.25 cm. Also, both showed the same specificity (80%), but size showed the highest sensitivity of 100% compared to wall thickness which showed a sensitivity of 75% (Figure 3).

**Table 5:** Sensitivity analysis

Test Result Variable(s)	AUC	P value	Cutoff	Sensitivity	Specificity	95% Confidence Interval	
Size	0.911	0.008	6.5 cm	100%	80%	0.810	1.000
Wall thickness	0.911	0.008	0.25 cm	75%	80%	0.789	1.000

The correlations between imaging findings and histopathology findings showed that the findings of ultrasound and CT showed significant association with the findings of histopathology (p=0.004, 0.016, for US and CT, respectively. On the other hand, there was no association found between MRI findings and histopathology findings (p=0.25) (Table 6).

**Table 6:** A paired comparison McNamara test

		Pathology				P value
		Benign		Malignant		
		Count	Row N %	Count	Row N %	
Ultrasound	Negative	27	100.0%	0	0.0%	0.004
	Positive	9	69.2%	4	30.8%	
CT	Negative	8	100.0%	0	0.0%	0.016
	Positive	7	63.6%	4	36.4%	
MRI	Negative	5	100.0%	0	0.0%	0.25
	Positive	3	100.0%	0	0.0%	

The comparison between US and CT is shown in Table 7. The sensitivity and negative predictive values of both modalities were the same (100%). CT showed a higher positive predictive value (36.36%) compared to the US (30.77%). On the other hand, the US showed higher specificity (75%) and accuracy (77.5%) compared to that of the CT scan; 53.33%, and 63.16 for specificity and accuracy, respectively.

**Table 7:** Comparison between US and CT Abbreviation: US, ultrasound; CT, computed tomography

	Ultrasound		CT scan	
Statistic	Value	95% CI	Value	95% CI
Sensitivity	100.00%	39.76% to 100.00%	100.00%	39.76% to 100.00%
Specificity	75.00%	57.80% to 87.88%	53.33%	26.59% to 78.73%
Positive Predictive Value	30.77%	20.15% to 43.90%	36.36%	24.96% to 49.53%
Negative Predictive Value	100.00%		100.00%	
Accuracy	77.50%	61.55% to 89.16%	63.16%	38.36% to 83.71%

## Discussion

**Figure 4:**
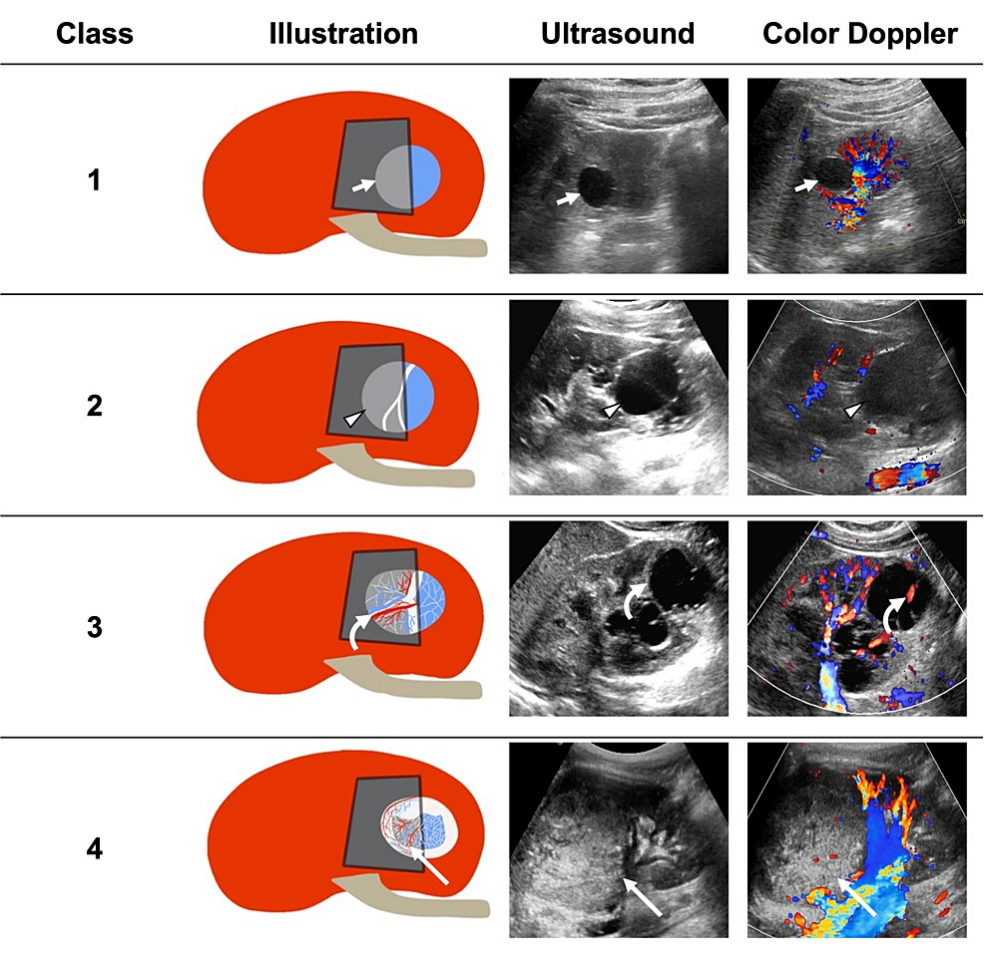
Modified Bosniak classification illustration mBosniak class 1, simple cyst with no flow in color Doppler (short arrows). mBosniak class 2, smooth wall and minimally complex thin septations with no flow in color Doppler (arrowheads). mBosniak class 3, thick or irregular septations with flow in color Doppler (curved arrows). mBosniak class 4, Solid or nodular component or thick irregular wall with flow in color Doppler (long arrows). Abbreviations: mBosniak, modified Bosniak Illustration created by the author MA.

Renal cysts are uncommon in the pediatric age group [14]. The incidentally detected renal cysts by US can cause frequent radiological and clinical dilemmas. Many children continue to be monitored for changes in the number and appearance of renal cysts [18]. The classification of renal cysts is based on the Bosniak classification, which was originally developed to classify renal cysts in adults. However, few studies conducted on the pediatric population suggested that the US-based modified Bosniak classification is reliable in classifying renal cysts in the pediatric population (Figure 4) [14,15].

Therefore, this study was conducted to apply the modified Bosniak classification system among children with renal cysts in a single center in Saudi Arabia.

Simple renal cysts are round, anechoic, thin-walled, non-septated, and separate from the collecting system [19]; whereas more complex renal cysts have soft tissue components or thick septation and can cause diagnostic dilemmas as they raise suspicions for the rare multilocular cystic nephroma and cystic nephroblastoma (Wilms tumor) [20].

Renal cystic disease consists of a broad spectrum of conditions that may lead to renal diseases. In children, a study included 237 pediatric patients; the study found that the largest proportion of patients had multicystic dysplastic kidney disease (47.25%), whereas 23.62% and 15.18% had autosomal dominant polycystic kidney disease and simple cyst, respectively. Further complications of patients who died were recorded including proteinuria (2.1%), chronic kidney injury (6.32%), and hypertension (4.21%). The authors revealed that renal cystic disease could lead to further complications, and periodic follow-up is necessary to avoid them. Also, early diagnosis is very important to prevent these complications and protect renal functions [2].

There were few studies that assessed the use of US, MRI, and CT and the use Bosniak classification system among children for the diagnosis of renal cysts. Grauman et al. conducted a study to compare the diagnostic accuracy of MRI, US, and CT for categorizing complex renal cystic masses according to the Bosniak classification. The authors demonstrated that CT should remain the gold standard of the Bosniak classification as MRI and the US up- and downgraded the renal cysts compared to CT [13]. A study by Peng et al. was conducted to compare contrast-enhanced multi-slice CT and US in the assessment of cystic renal disease among children based on the Bosniak classification. It was found that both modalities provided a highly accurate diagnosis for malignant renal cystic masses in children using the Bosniak classification system; however, the assessment of benign masses still needs improvement [21]. Another study assessed the incidentally detected renal cysts in children based on a modified Bosniak classification and found that this modified classification system could safely be used in the monitoring and management of incidentally detected simple renal cysts (categories I and II) in pediatric patients [16]. Another study was conducted to evaluate the correlations of the modified Bosniak categories assigned by radiologists to histological findings and inter-rater reliability with a focus on intermediate-risk lesions revealed that the implementation of the modified Bosniak classification in children caused a disconcerting underestimation of intermediate risk. This led to low inter-rater consistency for the categories intended to guide decisions regarding conservative and surgical management. The authors highlighted that clinicians should be cautious when using the modified Bosniak classification among children [22].

In our study, the characterization of the renal cysts among children showed that most cysts were well-defined, and most of them lacked irregularity of septations or internal vascularity. Few cysts showed calcifications and solid components, whereas septations (either thin or thick) were found among more than one-half of children. The modified Bosniak classification based on US showed that more than one-half of children were either in class I or II; however, this classification was changed based on CT and MRI, and fewer children were categorized in class I and II although histopathology was not performed for almost three-quarters of the children. Benign lesions were found among more than one-half of the samples of children on which histopathology was performed; only four children had Wilms tumor, which is a rare presentation [20].

It was suggested that the risk of transformation of simple renal cysts to renal malignancy in children is low [23], and this was in agreement with our findings based on radiological and histological findings.

The comparison between the three radiological modalities and histopathology findings showed that both US and CT showed a significant correlation with histopathology findings. Those who showed negative US or CT significantly reported benign lesions. On the other hand, MRI showed no significant correlation. Therefore, the diagnostic indices of US and CT were investigated. Both modalities showed the same sensitivity and negative predictive values, whereas CT showed higher positive predictive values; however, US showed much higher accuracy and specificity compared to CT. Therefore, US showed validity and more accuracy to be used in modified Bosniak classification for renal cysts among children. Also, US lacks the risk of radiation exposure exerted by CT.

Saltzman et al. conducted a study on 22 children patients with renal cysts detected by the renal US; they concluded that cystic renal lesions with modified Bosniak class I or II were most often benign, whereas class III or IV warranted surgical excision [15]. This was similar to our study, but we did not investigate the correlation between the histopathology of the lesions and the class reported by the US. However, negative findings of US and CT were associated with benign lesions. Another study also showed that simple and complex renal cysts of class I or II showed no malignancy in the surgical resection, and the US was sufficient for such a group [14].

A previous study by Kashgrai et al. revealed that the modified Bosniak classification could be safely used in monitoring and the management of incidentally detected simple renal cysts of class I and II in children [16]. However, the previous study did not compare CT and US, as in our study. US showed more accuracy and specificity for such lesions compared to CT.

Another study was conducted on children to compare contrast-enhanced CT (CECT) and US for the assessment of cystic renal masses using the Bosniak classification. It was found that the diagnostic accuracy of CECT was slightly better than in the US, but with no significant difference. Both US and CECT provided a highly accurate diagnosis for malignant renal cysts using the Bosniak classification, but benign masses still need improvement. Finally, the authors recommended that the US was the best screening modality in Bosniak I and II, whereas, in Bosniak III and IV, CECT is the first choice [21]. This was in agreement with our study, except that the US showed better accuracy and specificity compared to CT.

One study was conducted to examine the correlation of modified Bosniak categories assigned by radiologists to histological results and inter-rater reliability focusing on intermediate-risk lesions. The study showed that a correct classification was made in 41/56 imaging readings with a sensitivity of 73.2%. It was concluded that the implementation of modified Bosniak classification in children caused a disconcerting underestimation of intermediate risk. There was a low-inter-rater consistency for the categories included to guide decisions regarding conservative and surgical management. It was suggested to be cautious in using the modified Bosniak system for children [22]. However, the previous study was conducted on only seven children. Also, the study was limited by the inherent biases in a retrospective design, and the imaging protocols used were not standardized.

On the other hand, the modified Bosniak classification system was found to demonstrate good inter-observer agreement and identified the single tumor as a complex cyst. This study was a retrospective study but included a larger sample size (212 children). The vast majority of solitary renal cysts in children were simple and asymptomatic and did not require other imaging evaluation. Also, complex renal cysts were uncommon, and it was recommended to be evaluated with pre-intravenous and post-intravenous contrast CT. This was similar to our study [14].

In a study that compared the diagnostic accuracy of US, CT, and MRI used in Bosniak classification for renal cysts, it was found that contrast-enhanced US and MRI, both up- and downgraded renal cysts compared to CT and CT was advised to be the gold standard of the Bosniak classification. However, it should be noted that the previous study was conducted on the adult population aged 18 years and older [13]. This may explain the inaccuracy of MRI and US observed for Bosniak classification, whereas in our study among the pediatric population, US was more accurate and specific compared to CT. However, MRI was not a good diagnostic modality for Bosniak classification among children.

The histopathological findings in our study showed that it is significantly affected by the size, wall thickness, irregularity of septations, and solid components of the cysts. Malignant cysts significantly tended to be larger and have thicker walls, with irregular septations and solid components. Among these factors, both the size and wall thickness of cysts were assessed for diagnostic capability. Both factors showed the same AUC and specificity with the same p-value; however, the size of cysts had higher sensitivity compared to wall thickness.

## Conclusions

The modified Bosniak classification based on the US is sensitive, specific, and accurate for the classification of renal cysts (class I and II) among children. It is more accurate and specific compared to CT and lacks the risk of radiation exposure caused by CT. Also, the size of renal cysts can be a diagnostic marker of differentiation of benign and malignant cysts as it has high sensitivity and specificity at a cut of 6.5 cm.
